# The effect of physical activity level on the severity of diastolic dysfunction

**DOI:** 10.1186/s13102-023-00689-1

**Published:** 2023-06-30

**Authors:** Ali Ashraf, Siamak Rimaz, Abbas Seddighinejad, Amin karimi, Afagh Hassanzadeh-Rad, Mahboobeh Gholipour, Mahsa Motiei, Mohammad Ali Yazdanipour, Sheida Rimaz

**Affiliations:** 1 Clinical Research Development Unit of Poursina Hospital, Guilan University of Medical Sciences, Rasht, Iran; 2 Anesthesiology Research Center, Department of Anesthesiology, Alzahra hospital, Guilan University of Medical Sciences, Rasht, Iran; 3 Pediatric Diseases Research Center, Guilan University of Medical Sciences, Rasht, Iran; 4 Department of cardiology, Healthy Heart Research Center, Heshmat Hospital, School of Medicine, Guilan University of Medical Sciences, Rasht, Iran; 5 Student Research Committee, School of Medicine, Guilan University of Medical Sciences, Rasht, Iran; 6 Neuroscience Research Center, Guilan University of Medical Sciences, Rasht, Iran

**Keywords:** Exercise, Ventricular dysfunction, Echocardiography

## Abstract

**Background:**

Diastolic dysfunction (DD) is a risk factor for cardiovascular events in patients undergoing non-cardiac surgeries. Investigators aimed to assess the effect of physical activity level on the diastolic function of the left ventricle (LV) in patients attending the preoperative visit.

**Methods:**

This analytic cross-sectional study was conducted on 228 patients referred to Poursina hospital from November 2021 to March 2022. To define the physical activity level, we used the short form of the International Physical Activity Questionnaire (IPAQ). We categorized patients into inactive, minimally active, and health-enhancing physical activity groups. We also divided participants into three groups based on their daily sitting time. Also, echocardiographic parameters were calculated. The diastolic function of LV was evaluated, and its grading was defined from mild (grade1) to severe (grade 3).

**Results:**

Results showed that patients with DD had significantly higher age and lower levels of education (P < 0.001 and P = 0.005, respectively). After assessing echocardiographic parameters, we found that E/e’, TR Velocity, left atrial volume index, and pulmonary artery pressure had a statistically significant inverse relationship with physical activity level (P < 0.001 for all). Comparing physical activity level of subgroups showed that in HEPA (health-enhancing physical activity), the chance of developing grade 2 or 3 DD was reduced by 97% compared to the inactive group (OR = 0.03, P < 0.001). Still, there was no significant difference between the inactive and minimally active groups (P = 0.223).

**Conclusions:**

This study showed an inverse relationship between physical activity level and DD of the LV in a sample of 228 individuals attending the Anesthesia Clinic, independent of potentially confounding variables.Therefore, due to lower rate of DD in patients who are physically active, we can expect lower occurrence rate of cardiovascular events during surgery.

## Introduction

Heart failure (HF) is a chronic progressive problem with a poor prognosis in the long term, affecting more than 64 million people worldwide [1]. Diastolic dysfunction (DD) is the cause of HF in patients with preserved ejection fraction, and it can be a predictor of significant morbidity, hospitalization, cardiac death, and mortality [2]. On the other hand, a sedentary lifestyle, mainly accompanied by obesity, metabolic syndrome, and smoking, can induce insulin resistance and altered glucose metabolism that may be associated with an increased risk of cardiovascular diseases, such as DD [3, 4].

Current results showed that regular physical activity prevents DD and slows cardiac aging by improving left ventricular (LV) compliance [5]. Some previous studies also showed increased mortality in those who perform intense exercises [6]. However, there is a shortage of evidence on the reversed condition in which a sedentary lifestyle can develop DD. Notably, patients with DD are at risk of hypertensive crisis and pulmonary edema during the operation. Therefore, during preoperative echocardiography, anesthesiologists frequently visit asymptomatic patients with normal systolic function and abnormal diastolic parameters [7].

We assessed LV diastolic function by echocardiography in pre-operative visit due to its effects on patients’ prognosis during and after surgery. Considering the probable effect of physical activity level on cardiac diastolic function and its importance on health as well as the uncertainty regarding the effect of sedentary life style on this parameter, we aimed to determine diastolic function and its association with the amount of physical activity. For this purpose, we measured echocardiographic parameters of LV diastolic function. We identified its grade based on its latest classification in subgroups of healthy populations with different physical activity levels.

## Methods

### Study design

In this analytic cross-sectional study, patients referred to the anesthesiology clinic of Poursina hospital due to preoperative anesthesiology consultation, were assessed. This study was performed prospectively from November 2021 to March 2022. Patients were included by consecutive sampling method. Written informed consent letters were obtained from patients. The ethics committee of the Vice-Chancellor of Research at Guilan University of Medical Sciences approved this study (Code: IR.GUMS.REC.1399.303).

A checklist was designed to record demographic and clinical characteristics at the time of admission, including age, sex, level of education, marital status, alcohol consumption, smoking, hypertension, diabetes mellitus (DM), hyperlipidemia, cardiac medications, height, weight, body mass index (BMI), waist circumference (WC), hip circumference (HC), WC to HC ratio, and physical activity level.

To define the physical activity level, we used the short form of the International Physical Activity Questionnaire (IPAQ). It includes 27 open-ended questions about the duration (minutes) and frequency (days per week) of walking and any moderate to vigorous-intensity activity for at least ten continuous minutes in leisure and domestic time, working and transporting during a regular week (Excluding the week waiting for surgery, traveling, etc.). We assessed physical activity by scoring it in metabolic equivalent of task (MET)-minutes (min) to categorize patients. They were classified into inactive, minimally active (at least 600 MET-min/week), and health-enhancing physical activity (HEPA active with at least 3000 MET-min/week) groups based on the IPAQ classification [7]. To evaluate sedentary behaviors, we asked how long patients spend in a sitting position per day (dividing them into three groups: <5, 5–9, ≥ 10 h/day).

### Participants

Patients with left ventricular ejection fraction (LVEF) < 50%, hypertrophic or dilated cardiomyopathy, ischemic cardiomyopathy, valvular replacement or valvular surgery history, severe valvular disease, cardiovascular disease, history of rheumatologic or musculoskeletal diseases, history of malignancies, consistent arterial fibrillation, and congenital heart disease were excluded. Sample size was noted by G- power analysis with 0.05 α and 80% power as 228 participants in each group. The effect size d for this sample size was indicated as 0.2624723.

### Measurements

Nurses measured height by a tape meter (Seca, Germany) while patients stood with heads and heels touching the wall surface without shoes. Weight was measured by a calibrated digital scale (Seca, Germany) without heavy clothes or shoes. Blood pressure was measured when participants were comfortable with their right hand at heart level using an automated oscillometric device (Glamor, Germany). Hypertension was defined as systolic blood pressure 140 mmHg or higher, diastolic blood pressure 90 mmHg or higher, or routine use of antihypertensive medications. WC was measured by wrapping a tape meter around patients’ waist, in the middle line between anterior superior iliac spine and the last rib, after they breathed out [8], and HC was determined by measuring around the largest part of buttocks. Body surface area (BSA) was calculated by Dubois formula: BSA = 0.20247 [weight 0.425 × (height/100)0.725] [9].

### Echocardiography

All participants underwent an echocardiography examination performed by an experienced cardiologist using Samsung HS70A Ultrasound Machine. We made triplicate measurements for each parameter in sinus rhythm in patients. LV volumes and LVEF were calculated with the modified biplane Simpson’s method using two-dimensional (2D) images from the apical four- and two-chamber views. We measured the left atrial diameter in an anteroposterior view as well.

We measured intraventricular septum thickness (IVST), Left posterior wall thickness (LPWT), and diameter of the LV cavity using the linear 2D method from the parasternal long-axis view at the end of systole and diastole. Relative wall thickness (RWT) was calculated by two times PWT divided by the diastolic diameter of LV. LV mass was calculated in grams from the linear dimensional of LV and then indexed in BSA, which is defined as LV mass index (LVMI).

The diastolic function of LV was evaluated in an apical four-chamber view assessing transmitral LV inflow. E-wave (early peak of filling velocity) and A-wave (late peak of filling velocity) during atrial systole and deceleration time of E velocity were noted. Tissue Doppler imaging was performed to obtain transmitral annular velocities (lateral e’, lateral a’, septal e’, and septal a’) in cm/s. The Dubois formula calculated LV mass.

DD grading was defined from mild (grade1) to severe (grade 3) based on the latest update from the American Society of Echocardiography and the European Association of Cardiovascular Imaging [10].

### Statistical analysis

Data were presented as number, percent, mean, and standard deviation (SD). SPSS Statistics analyzed them for Windows, version 16. 0 (SPSS Inc., Chicago, Ill., USA). The Shapiro-Wilk test was used to check the normality of the quantitative variables. To compare groups and assess the relationship between them, after determining normality, we used T-test if it followed the normal distribution and the Mann-Whitney U test if it was non-normally distributed. Chi-square and Fisher’s exact tests were used to compare the qualitative variables. We also applied the regression analysis by the backward LR to indicate the predictors. A ROC curve (receiver operative characteristic curve) was designated to define cut-off points for anthropometric indices in DD. The significance level in all tests was considered 0.05.

## Results

Two hundred twenty-eight patients with a mean age of 53.46 ± 13.43 years were enrolled in this study. Based on the nature of our study we had no missing data. The majority of patients were females (62.28%), married (92.98%), educated for 12 years or less (78.51%), and did not have a history of smoking (88.16%) or alcohol consumption (93.86%). Results also showed that most patients were obese, with a mean waist to pelvic ratio of 0.93 ± 0.11. A total of 193 (88.88%) participants had DD, with most (55.44%) having grade 1 DD. To define cut-off points for anthropometric indices in DD, our results showed that none of the variables were significant (P > 0.05). Therefore, we didn’t mention cut-off points, sensitivity, and specificity of them (Table 1).

**Table 1:** ROC curve analysis for anthropometric indices

Variable	AUC	95% CI		P-value
		Lower	Upper	
BSA	0.451	0.350	0.553	0.359
WC/HC	0.583	0.470	0.696	0.119
WC	0.585	0.475	0.696	0.109
Weight	0.509	0.402	0.615	0.869
Height	0.414	0.321	0.506	0.104
BMI	0.553	0.449	0.658	0.315

AUC: Area under the curve, CI: Confidence interval, BSA: Body surface area, WC: Waist circumference, HC: Hip circumference, WC/HC: Waist circumference/ Hip circumference, BMI: Body mass index

In Table 2, the findings showed that participants with DD were mainly male, married, older, less educated, current or past smokers, nonalcoholic, and obese. Results showed that patients with DD had significantly higher age and lower levels of education (P < 0.001 and p = 0.005, respectively).

**Table 2:** Baseline demographic and clinical characteristics of participants according to their diastolic function

			Normal diastolic function				Diastolic dysfunction				P-Value		
			No.		%		No.		%				
Age group (years)	> 45		26		45.61		31		54.39		< 0.001*		
		45–65		9		6.98		120		93.02			
		≤ 65		0		0.00		42		100.00			
		Total		35		15.35		193		84.65			
		Mean ± SD		39.29 ± 9.97				56.04 ± 12.34				< 0.001***	
Sex		Male		9		10.47		77		89.53		0.111*	
		Female		26		18.31		116		81.69			
		total		35		15.35		193		84.65			
Marital status		Unmarried		5		31.25		11		68.75		0.078**	
		Married		30		14.15		182		85.85			
		Total		35		15.35		193		84.65			
Level of education		≤ 12 years		25		13.97		154		86.03		0.268*	
		> 12years		10		20.41		39		79.59			
		Total		35		15.35		193		84.65			
Smoking		No		32		15.92		169		84.08		0.402**	
		Past smoker		1		5.56		17		94.44			
		Yes		2		22.22		7		77.78			
		Total		35		15.35		193		84.65			
Alcohol consumption		No		32		14.95		182		85.05		0.456**	
		Yes		3		21.43		11		78.57			
		Total		35		15.35		193		84.65			
BMI		Normal		10		18.52		44		81.48		0.730*	
		Overweight		10		15.38		55		84.62			
		Obese		15		13.76		94		86.24			
		Total		35		15.35		193		84.65			
		Mean ± SD		28.05 ± 5.87				29.16 ± 5.39				0.270***	
WC to HC ratio		Mean ± SD		0.91 ± 0.10				0.93 ± 0.11				0.236***	
Diabetes		No		34		18.99		145		81.01		0.004*	
		Yes		1		2.04		48		97.96			
		Total		35		15.35		193		84.65			
Hypertension		No		28		23.73		90		76.27		< 0.001*	
		Yes		7		6.36		103		93.64			
		Total		35		15.35		193		84.65			
Hyperlipidemia		No		31		21.09		116		78.91		0.001*	
		Yes		4		4.94		77		95.06			
		Total		35		15.35		193		84.65			
Cardiac medication		No		26		27.66		68		72.34		< 0.001*	
		Yes		9		6.72		125		93.28			
		Total		35		15.35		193		84.65			

* Chi-square test

** Fischer exact test

*** Independent T-test

BMI: Body mass index, WC: waist circumference, HC: Hip circumference

Coronary artery disease (58.77% used cardiac medication) and hypertension (48.25%) were the most prevalent underlying diseases. There was also a significant association between DD and DM, hypertension, hyperlipidemia, and cardiac medication use (P = 0.004, P < 0.001, P = 0.001, P < 0.001, respectively).

Table 3 shows that most patients had ≥ 10 h of sitting time (53.51%). Participants with inactive and minimally active physical activity level had the highest percentage of DD. Although there was a statistically significant association between DD and physical activity level (P < 0.001), sitting time and DD were not significantly associated (P = 0.588).

**Table 3:** Prevalence of DD in participants according to their physical activity level and sitting time

		Normal diastolic function		Diastolic dysfunction		Total		P-Value
		No	%	No	%	No	%	
Sitting time	< 5 h (hrs)	5	16.67	25	83.33	30	13.16	0.588*
	5–10 h	14	18.42	62	81.58	76	33.33	
	≥ 10 h	16	13.11	106	86.89	122	53.51	
	Total	35	15.35	193	84.65	228	100.00	
	Mean ± SD	9.11 ± 4.40		11.16 ± 6.37		10.85 ± 6.15		0.070**
	(Lowest, Highest)	(3.0, 20.0)		(1.0, 40.0)		(1.0, 40.0)		
Physical activity	Inactive	1	2.22	44	97.78	45	19.74	< 0.001*
	Minimally active	1	2.70	36	97.30	37	16.23	
	HEPA	33	22.60	113	77.40	146	64.04	
	Total	35	15.35	193	84.65	228	100.00	

* Chi-square test

** Independent T-test

Assessing echocardiographic parameters showed that E/e’, TR Velocity, left atrial volume index (LAVI), and pulmonary artery pressure (PAP) had a statistically significant relationship with physical activity level. As physical activity level decreased, these indicators increased (P < 0.001 for all) (Table 4).

**Table 4:** Echocardiographic parameters associated with diastolic function of LV in three subgroups of physical activity level

	Level of physical activity median(IQR*)			
	Inactive	Minimally active	HEPA	P-Value**
LVEF	55.00(55.00–55.00)	55.00(55.00–55.00)	55.00(55.00–55.00)	0.630
BSA	1.89(1.79–2.03)	1.76(1.64–1.96)	1.78(1.66–1.90)	0.003
LVM	131.00(96.00-155.00)	142.00(114.00-185.00)	120.50 (93.00-163.00)	0.108
LVMI	65.00(52.00–83.00)	71.00(64.00-95.60)	68.00(52.00–88.00)	0.118
RWT	0.36(0.33–0.40)	0.38(0.33–0.44)	0.37(0.33–0.40)	0.605
E/e’	14.00(14.00–15.00)	14.00(10.00–14.00)	9.00(8.00–10.00)	< 0.001
TR velocity	2.80(2.70–2.90)	2.70(2.60–2.80)	2.30(2.10–2.50)	< 0.001
LAVI	36.00(34.00–36.00)	35.00(32.00–36.00)	30.00(29.00–30.00)	< 0.001
PAP	30.00(30.00–33.00)	30.00(29.00–32.00)	24.00(20.00–26.00)	< 0.001

*IQR: Interquartile Range

**Kruskal Wallis test

LV-EF: Left ventricular ejection fraction, BSA: Body surface area, LVM: Left ventricular mass, LVMI: Left ventricular mass index, RWT: Relative wall thickness, TR: Tricuspid regurgitation, LAVI: Left atrial volume index, PAP: Pulmonary artery pressure

After adjusting confounders in Backward logistic regression, there was still a significant relationship between physical activity level and DD (P = 0.001). In HEPA, compared to the inactive group, the chance of developing grade 2 or 3 DD was reduced by 97% (OR = 0.03, P < 0.001), but there was no significant difference between the inactive and minimally active groups (P = 0.223). Age was also significantly associated with grading of DD. Based on the mentioned OR (1.04), by each year of aging the relative chance of developing DD was 4%, therefor we can expect 20% increase in this chance by 5 years of aging (P < 0.030) (Table 5).

**Table 5:** Relationship between physical activity and any Grade of DD using a multivariate logistic regression

	Coefficients	Standard error	P-Value	Odds ratio(OR)	95% CI OR	
					Lower limit	Higher limit
Age(years)	0.037	0.017	0.030	1.038	1.004	1.073
Level of physical activity			0.000			
Minimally Active/Inactive	− 0.743	0.610	0.223	0.476	0.144	1.572
HEPA/Inactive	-3.589	0.533	0.000	0.028	0.010	0.078
Constant number	− 0.086	0.964	0.929	0.917		

HEPA: Health enhancing physical activity

## Discussion

Anesthesiologists usually focus on the systolic function of LV and cardiac wall characteristics in preoperative risk assessment. However, due to instability of hemodynamic status during anesthesia, patients with DD are more prone to develop cardiovascular events and pulmonary edema in non-cardiac surgeries [8]. As this was the first study assessing the association between physical activity level and the grading of DD and its parameters, our promising results showed that clinicians should consider physical activity level as a contributing factor to the development of DD during the pre-operation visit.

In a previous study, the inverse association between DD and physical activity was also reported by using only a simple question to determine physical activity level, which divided people into active and inactive groups [5]. Our investigation used IPAQ as a validated international method with 27 open-ended questions to score physical activity level. DD grading was performed based on the latest classification update from the American Society of Echocardiography and the European Association of Cardiovascular Imaging [10]. In previous studies, physical activity level had been associated with developing DD. So, we assessed the association of physical activity level with each LV diastolic function parameter to assess their association and pathophysiology better.

LV mass indexed to BSA and yielded the LVMI [11]. Besides, we found that patients with low physical activity level had a higher BSA. Echocardiographic parameters, including E/e’, TR Velocity, LAVI, and PAP, had a statistically significant inverse relationship with physical activity level. We explored the association between echocardiographic parameters and physical activity in previous studies. A study by Patel et al. in 2021 on 3433 participants showed that higher baseline cardiorespiratory fitness (CRF) and BMI were associated with higher tricuspid annular systolic velocity [12]. Another study by Pandey et al. supported the inverse association between CRF and E/e‘[13]. Berdy et al. in 2021 expressed the positive association between physical activity and cardiac function by showing the association between higher physical activity and lower LVMI and E/e’ in participants [14].

LAVI increases when DD happens because the impaired diastolic function of LV causes remodeling of the left atrium (LA), resulting in increased LA pressure to maintain adequate blood filling in LV [15]. In contrast, LA enlargement may be a physiological response to exercise. It raises the need for more stroke volume, puts pressure on cardiac walls, and induces increased size in cardiac chambers. The positive relationship between physical activity and LAVI was supported in a recent cross-sectional study only in individuals with normal diastolic function [16]. In Bjerring et al., on preadolescent athletes, no association between exercise and LAVI was reported [17]. Therefore, the inverse relationship between LAVI and physical activity level in our nonathletic population was notable.

In agreement with Ryu et al. [12], we found that sitting time and DD were not associated, supporting the idea that sitting time may not be a reliable indicator of a sedentary lifestyle. It may be because people hardly remember how much time they spend in a sitting position rather than the time they spend doing physical activity.

We assessed individual-contextual factors that could be associated with DD in our sample and found that age was the only factor associated with developing higher grade of DD. ArbabZadeh et al. also showed this association between age and DD by emphasizing that prolonged endurance exercise can prevent the mechanisms of DD that occur by aging [2].

In justifying the findings of various studies, including our research, it can be said that long-term endurance exercises lead to enlargement of ventricular volume and dimensions, leading to improved cardiac function without significant changes in contractility. In addition, long-term exercise’s beneficial effects can manifest in maintaining vascular tension, reducing arterial pressure, and maintaining the elasticity of blood vessels even by getting older. Therefore, changes in cardiac myocyte morphology, a physiological response to arterial changes in elders, will be less apparent in those who are physically active. However, the exact mechanism of LV diastolic disorder and its inverse relationship with physical activity has not been identified yet, and the mentioned materials remain at the hypothesis level.

In justifying the findings of this study, since obesity is related to the structural and functional changes of the heart, and as we expect less activity in obese people, it may indicate the inverse association between the possible mechanism of higher physical activity level and the occurrence of LVDD. However, our study surprisingly showed no significant relationship between BMI and DD.

Insulin resistance is another possible mechanism that can cause DD in inactive individuals. Several studies have shown that exercising is associated with improving insulin sensitivity. In reverse conditions, a sedentary lifestyle increases body fat contributing to insulin resistance. Ayalon et al. and Hwang et al. showed an association between insulin resistance and DD [18, 19]. In our investigation, hyperlipidemia and DM were significantly associated with the DD of LV. Although in the regression analysis, we missed the significant relationship between LVDD with DM and hyperlipidemia, in the study of Ryu et al., even after adjusting for the effect of DM, the relationship between physical activity level and LVDD was maintained [12]. It indicated the possible direct effect of physical activity from biological pathways independent of obesity or insulin resistance on LV diastolic function.

The justification of a positive relationship between physical activity and LVDD may be explained by high blood pressure. In the current study, a statistically significant association was observed between DD and hypertension; therefore, the percentage of DD was higher in people with hypertension. Additionally, spending less time in sedentary activities and more time in light activities can lower blood pressure in hypertensive individuals [20]. Consistent with the results of our study, the relationship between high blood pressure and LVDD has been proven in Erdogan et al.‘s study [21]. Still, the results of Backward-LR regression showed no significant relationship between hypertension, and the LVDD contradicts the results of the study by Ryu et al. [12]. Even in the regression analysis, there was a relationship.

### Limitations

This study had some limitations. First, the report of physical activity level was self-reported and measurement error is inevitable in this study. In particular, people usually have difficulty remembering exercises with light intensity and tend to have recall bias on the amount of time spent in such activities. Therefore, the actual association of physical activity level with LV diastolic function could be much stronger than the values ​​obtained in this study. The reduced chance of developing grade 2 or 3 DD in HEPA group may be affected by age. Also, the cross-sectional design in this study led to a decrease in our ability to indicate causal relationships, and our sample size may not represent the general population. Moreover, quantitative data were non-normally distributed, therefore, we used nonparametric Kruskal-Wallis test and compared groups based on median and interquartile range. As we examined patients who were candidate for non-cardiac surgeries and we were unaware of the exact type of surgery, we could not compare the results in different types of surgery. Another limitation of the present study was the lack of statistical comparison of the impact of physical activity level on LVDD in men compared to women. Although the rate of LVDD was not significantly different regarding sex, we did not assess the relationship between DD and physical activity level based on sex. Therefore, we suggest future studies exploring this issue. Besides, we recommend performing further studies assessing the relationship between post-operative outcomes and physical activity level.

## Conclusion

This study showed an inverse relationship between physical activity level and DD of the LV in a sample of 228 individuals attending the Anesthesia Clinic, independent of potentially confounding variables. Our results indicated that the increased physical activity level might be independently associated with a reduced LV resting period disorder risk. Therefore, due to lower rate of DD in patients who are physically active, we can expect lower occurrence rate of cardiovascular events during surgery.

## Data Availability

The datasets used and/or analyzed during the present study are available from the corresponding author on reasonable request.

## Abbreviations

DD: Diastolic Dysfunction
LV: Left Ventricular
IPAQ: International Physical Activity Questionnaire
HF: Heart Failure
DM: Diabetes Mellitus
BMI: Body Mass Index
WC: Waist Circumference
HC: Hip Circumference
MET: Metabolic Equivalent of Task
Min: Minute
HEPA: Health-Enhancing Physical Activity
LVEF: Left Ventricular Ejection Fraction
BSA: Body Surface Area
2D: Two-Dimensional
PAP: Pulmonary Artery Pressure
IVST: Intraventricular Septum Thickness
LPWT: Left Posterior Wall Thickness
RWT: Relative Wall Thickness
LVMI: Left Ventricular Mass Index
ROC: Receiver Operative Characteristic
SD: Standard Deviation
LAVI: Left Atrial Volume Index
LA: Left Atrium
CRF: Cardiorespiratory Fitness
